# The Week's Work

**Published:** 1910-12-03

**Authors:** 


### The Week's Work.

### EMINENT CHAIRMAN SERIES.

We regret that owing to exigencies of space we are forced to keep over the monthly article on Eminent Hospital Chairmen this week. The next article in the series, which will deal with the work of Mr. Cosmo Bonsor at Guy's Hospital, will be published in next week's issue of The Hospital. It will be remembered that the first article of the series appeared in our issue of October 1, page 25.

### A NEW MEDICAL SUPERINTENDENT FOR THE HOPE HOSPITAL, SALFORD.

The President of the Local Government Board having firmly refused to sanction the recent appointment of a medical superintendent at the Hope Hospital, Salford, which the Guardians had filled—as The Hospital pointed out on September 10, page 714, in contravention of the conditions of their own advertisement—a new appointment has been made. This, we understand, gives to Mr. E. E. Chambers Austin the post for which forty-seven applications had been received, and awaits only ratification by the Local Government Board.

### THE PARK HOSPITAL FOR CHILDREN.

On Saturday, November 19, Mr. Burns opened the Park Hospital for Sick Children at Hither Green, S.E. Elsewhere in this issue we give a brief description of the adapted hospital, together with copies of the plans, which show that the institution is in every way suitable for the purpose which has now been assigned to it. A children's hospital is a necessity in the part of London where this building stands, and those interested in the hospital expansion of the metropolis will follow the fortunes of the Park with close attention during the next few years. Mr. Burns, in opening the hospital, referred to the excellent work that such institutions are doing on behalf of our child population; it is a subject to which the attention of the public cannot be too frequently drawn. The adult hospital population of every large city is inevitably recruited from the mass of sick children who are untreated or carelessly treated because proper facilities for their reception in hospital are not available. It is not too much to say, with a well-known Munich professor of pediatrics, that this hospital population is abnormally large in cities which possess very few or no children's hospitals. The statistics on this subject have not yet been worked out, but a preliminary glance at the figures given for towns in which the hospital accommodation for children is not what it ought to be seems to support this presupposition. It is of the greatest importance, therefore, that the public should be made aware of the fact that properly equipped children's hospitals are a vital necessity in every large town. It is becoming more and more generally accepted that the addition of children's wards to a general hospital, while it helps considerably, can never obviate the necessity of providing such special hospitals for children in any district where their want is felt.

### THE LARGEST CHILDREN'S HOSPITAL IN THE WORLD.

Special interest attaches to the Park Hospital. It is not entirely an experiment. The Children's Infirmary at Carshalton, which was opened for the reception of juvenile patients early in 1909, was the real trial attempt, made without fuss or ostentation, so quietly, in fact, that the public is probably not aware that the Metropolitan Asylums Board is already supporting a children's hospital, and that at no great distance from London there exists a State-aided institution which is a general hospital just as much as the Shadwell or Great Ormond Street Hospital is. At Carshalton, since the institution was opened, some 4,000 child-patients have been treated, their ages ranging from infancy to fourteen years of age. They have suffered from all sorts of diseases. The surgical department, for instance, has been kept busy with cases of tubercular osteitis and arthritis, diseases of the bones, ears, eyes, nose, and throat, and of the digestive and respiratory systems, not to mention deformities, while the medical department has attended to the whole gamut of infantile disorders falling more particularly to its charge. Carshalton is the largest children's hospital in England; the Park Hospital will be the second largest, and both these institutions will therefore be able to claim the proud position of being the largest of their kind in the world until the new Munich Children's Clinic is completed. Compared with them, Great Ormond Street, with its 230 beds, is a pigmy, and even the large Continental and American special institutions for children seem small.

### WHAT THE PARK HOSPITAL MEANS.

The results of this new departure are likely to be significant. No voluntary hospital can compete with the Asylums Board, backed as the latter is by State support. The attraction of so much material is bound to influence medical men who are specially interested in pediatrics, just as the advantages of being attached to a fever hospital have weighed with those who were specially interested in the study of infectious diseases. Whatever one may say about the management of the fever hospitals it is certain that a very large amount of excellent research work has been done in them; work which it would have been absolutely impossible to have achieved in voluntary hospitals. It is well known that fever specialists are men who are attached to these large Asylums Board hospitals; is it too much, therefore, to contemplate in the near future the decline of our small children's hospitals and the rise of the Asylums Children's Hospital, with the concomitant creation of a staff of highly expert pediatrists who, from the facilities granted them at such hospitals, their comparative independence and their secured future as Government officials, are bound to make headway at the expense of their colleagues, who are free lances in the voluntary hospitals? No one will deny that the establishment of such hospitals as Carshalton and the Park opens a wide vista of possibilities for the future. Whether the outlook is a bright or a gloomy one depends largely on the view we individually take with regard to the advisability of the Asylums Board competing on such a large scale with the general hospitals. But the fact must be admitted: these new institutions have been established. And those who are really concerned about the welfare of the child population in London will probably agree with us that both Carshalton and the Park Hospital will do an immense amount of good in alleviating the suffering and distress among workhouse and infirmary children in their respective neighbourhoods.

### HOSPITALS IN THE CAPE STATE.

The latest official report of the hospitals and asylums of the Cape Colony deals with the state of affairs existing in 1909 and shows several points of interest. For instance, the following details about the proportionate payments for expenditure from various sources is instructive in view of the fact that the hospitals in the Colony are to a large extent State-supported.

| | Government. | Paying Patients. | Local Bodies and Public. |
|---|---|---|---|
| 1909 ... ... ... | 45.70 | 21.58 | 18.84 |
| 1908 ... ... ... | 46.26 | 19.46 | 16.10 |

From this return it is evident that the payments from local and public bodies and from paying patients are steadily increasing—a matter of some importance when one considers that comparative figures in Australia show a falling-off in the contributions from those sources since the State has actively interested itself in hospitals. There are one or two points in the report which should be explained. For example, a hospital (Umtata) which possesses only seventeen beds gives a return of 18.01 patients as its "daily average." This institution has therefore evidently solved the riddle of Numenius, "How to place ten people in a chamber in which only five can go." Or possibly it has merely imitated the French hospitals and offered "hospitality" to so many patients in excess of its reasonable complement! A very satisfactory point is the decrease in the average daily cost per patient, which is, for all the hospitals, 6s. 1d. per day.

### A HOSPITAL AS SUCCESSFUL LITIGANT.

St. Thomas's Hospital may congratulate itself on Mr. Justice Eve's ruling, by which certain property under the will of Mrs. Harding passes to the institution and not to her next-of-kin. Mrs. Harding died in August 1909, and by her will directed that the residue of her effects should be sold and that the proceeds, "together with all moneys deposited or invested in any banks or institutions or owing or due to me at the time of my decease except as hereinbefore provided for be paid to the Governors of St. Thomas's Hospital." The testatrix died possessed of a considerable sum of Consols, and it was argued on behalf of the next-of-kin that Consols were not moneys deposited or invested in any bank or institution or owing or due to the testatrix. In giving judgment in favour of the hospital Mr. Justice Eve said it was clear that the testatrix intended to dispose of the whole of her property, and that she intended the hospital to take the residue of her estate. But then it was argued forcibly that Consols were not moneys deposited or invested in an institution or owing or due to her at her death. His Lordship, however, thought it was quite possible that the testatrix thought the money *was* invested in an institution—an institution, Mr. Justice Eve went on, which still happily existed, and in which we were all interested. The hospital, therefore, was entitled to the whole of the residue. This verdict will be satisfactory to institutional workers everywhere, if only on the general ground that in cases in which one party is an individual and the other a corporate body, the corporate body is thought generally to have little chance of a favourable verdict. That opinion, however, applies only to cases tried by jury, and has been urged, indeed, as an argument against the jury system; but in the present case, heard in the Chancery Division of the High Court, of course it cannot be brought forward. The success of the hospital in this case will not, we hope, make hospital treasurers in general any less careful and considerate in providing and making known to possible benefactors forms of bequest concerning the legal interpretation of which there can be no question.

### THIS WEEK'S DRUG MARKET.

The advance in the cost of glycerine, which was stated in last week's report to be imminent, has taken place; the price has never been higher than it is at present, and it is not improbable that a further advance will take place before the end of the winter. Seidlitz powder and tartarated soda have also been raised in price again, as a consequence of the limited supply of cream of tartar. Ergot of rye is still tending dearer. Oil of cloves has again been slightly advanced in price. High values continue to be quoted for gum benzoin. Belladonna root is scarce and dearer. Jalap is slightly cheaper. The price of opium is well maintained. Another diminution in the price of quicksilver is not at all unlikely. Business in drugs continues good.

